# Consensus document on electroencephalography education in anaesthesiology: defining learning outcomes: A modified four-round Delphi study

**DOI:** 10.1097/EJA.0000000000002346

**Published:** 2026-02-19

**Authors:** Joana Berger-Estilita, Dana Baron Shahaf, Odmara L. Barreto Chang, Vincent Bonhomme, Iulia Crisan, Juan L. Fernández-Candil, Paul S. Garcia, Mia Gisselbaek, Bryan A. Glezerson, Darren Hight, Karina Jakobsen, Ivan Jonassen Rimstad, Heiko A. Kaiser, Stephan Kratzer, Susanne Koch, Ozlem Korkmaz Dilmen, Matthias Kreuzer, Janna Helfrich, Friedrich Lersch, Francisco A. Lobo, Adrian P. Marty, Isabel Marcolino, Başak C. Meco, Ben Palanca, Stefanie Pilge, Finn M. Radtke, Frank Rasulo, Aeyal Raz, Stefano Romagnoli, Carolina S. Romero, Gerhard Schneider, Pablo O. Sepúlveda, Daniel Shmukler, Barbara Sinner, Martin Soehle, Felipe S. Thyrso de Lara, Susana Vacas, Sérgio Vide, Falk von Dincklage, Sarah Saxena, Anthony R. Absalom

**Affiliations:** From the Institute for Medical Education, University of Bern (JBE), Department of Anaesthesiology and Intensive Care, Salem-Spital, Hirslanden Hospital Group, Bern, Switzerland (JBE), RISE-Health, Centre for Health Technology and Services Research (CINTESIS), Faculty of Medicine, University of Porto, Porto, Portugal (JBE), Department of Anaesthesiology and Intensive Care, Rambam Healthcare Campus, Haifa, Israel (AR, DBS), Department of Anesthesia and Perioperative Care, University of California San Francisco, San Francisco, California, USA (OLBC), Department of Anaesthesia and Intensive Care Medicine, Liège University Hospital, Liège, Belgium (VB), Anaesthesia and Perioperative Neuroscience Laboratory, GIGA-Consciousness, GIGA-Research, Liège University, Liège, Belgium (VB), Department of Intensive Care Medicine, University Hospital of Zürich, Zürich, Switzerland (IC), Department of Anaesthesiology and Surgical Intensive Care, Hospital del Mar; Hospital del Mar Research Institute, Barcelona, Spain (JLF-C), Department of Anesthesiology, Columbia University Medical Center, New York, New York, USA (PSG), Division of Anaesthesiology, Department of Anaesthesiology, Clinical Pharmacology, Intensive Care and Emergency Medicine, Geneva University Hospitals and Faculty of Medicine (MG), Unit of Development and Research in Medical Education (UDREM), Faculty of Medicine, University of Geneva, Geneva, Switzerland (MG), Department of Anesthesiology, Montréal Neurological Institute and Hospital, McGill University, Montréal, Québec, Canada (BAG), Department of Anaesthesiology and Pain Medicine, Inselspital, Bern University Hospital, University of Bern, Bern, Switzerland (FL, HAK, DH), Department for Anesthesiology and Intensive Care, School of Medicine, Technical University of Munich, Munich, Germany; and Hessing Clinic for Anesthesiology, Intensive Care and Pain Medicine, Augsburg, Germany (SK), Department of Anaesthesiology and Intensive Care, Zealand University Hospital, Nyk⊘bing F., Denmark (SK, KJ), Department of Anesthesiology, University of Minnesota Medical School, Minneapolis, MN, USA (FMR), Department of Anaesthesiology, Oslo University Hospital, Oslo, Norway (IJR), Centre for Anaesthesiology and Intensive Care, Hirslanden Klinik Aarau, Hirslanden Medical Group, Aarau, Switzerland (HAK), Charité - University Medicine Humboldt University Berlin, Department for Anesthesiology and Intensive Care Medicine, Berlin, Germany (SK), Istanbul University-Cerrahpasa, Cerrahpasa Faculty of Medicine, Department of Anesthesiology and Intensive Care, Istanbul, Turkey (OKD), Department of Anaesthesiology and Intensive Care, School of Medicine and Health, Technical University of Munich, Munich, Germany (GS, SP, MK), Department of Anesthesiology, Yale School of Medicine, New Haven, Connecticut, USA (JH), Anesthesiology and Pain Medicine Institute, Sheikh Khalifa Medical City, Abu Dhabi, United Arab Emirates (FAL), Balgrist University Hospital, Zurich (APM), Institute of Anaesthesiology and Intensive Care, Spital Limmattal, Schlieren, Switzerland (IM), Anaesthesiology and Intensive Care Unit Department, Ankara University Faculty of Medicine (BCM), Ankara University Brain Research Center (AÜBAUM); Department of Interdisciplinary Neuroscience, Health Sciences Institute, Ankara University, Ankara, Turkey (BCM), Department of Anesthesiology, Washington University School of Medicine, Saint Louis, Missouri, USA (BP), Department of Neuroanesthesia, Neurocritical and Postoperative Care, Spedali Civili University Hospital of Brescia, Brescia (FR), Department of Anesthesia and Critical Care, Azienda Ospedaliero-Universitaria Careggi, Florence, Italy (SR), Department of Health Science, University of Florence, Florence, Italy (SR), Department of Anaesthesia, Intensive Care and Pain Medicine, General University Hospital. Universidad Europea de Valencia, Valencia, Spain (CSR), Hospital Base San José Osorno, Universidad Austral de Chile (POS), Private practice, Johannesburg, South Africa (DS), Department of Anaesthesia and Intensive Care Medicine, Medical University Innsbruck, Innsbruck, Austria (BS), Department of Anaesthesiology and Intensive Care Medicine, University Hospital Bonn, Bonn, Germany (MS), Metropolitan University of Santos (UNIMES), Santos, Brazil (FT), Irmandade da Santa Casa da Misericórdia de Santos Hospital, Santos, Brazil (FT), Department of Anesthesiology, Mass General Brigham, Harvard Medical School, Boston, Massachusetts, USA (SV), Anaesthesiology Department, Unidade Local de Saúde de São João (SV), UNIC@RISE, Centre for Health Technology and Services Research, Faculty of Medicine, University of Porto, Porto, Portugal (SV), University Medicine Greifswald, Department of Anaesthesia, Intensive Care, Emergency Medicine and Pain Medicine, Greifswald, Germany (FvD), Department of Anaesthesiology, Helora (SS), Department of Surgery, UMons, Research Institute for Health Sciences and Technology, University of Mons, Mons, Belgium (SS), and Department of Anaesthesiology, University Medical Center Groningen, University of Groningen, Groningen, The Netherlands (ARA)

## Abstract

**BACKGROUND:**

The brain is the primary target of general anaesthesia, yet routine brain monitoring remains underutilised. Although electroencephalography (EEG) is increasingly recognised as an important tool for assessing brain states during anaesthesia, inconsistent training and over-reliance on processed indices have hindered its adoption in clinical practice.

**OBJECTIVE(S):**

To define a set of core learning outcomes for EEG education in anaesthesiology through expert consensus, aiming to support the development of a structured, tiered curriculum for both basic and advanced EEG training.

**DESIGN:**

Modified four-round Delphi study.

**SETTING:**

Multinational, online Delphi panel involving 33 anaesthesiology experts across various academic and clinical institutions. The study was conducted between 29 May 2023 and 28 November 2024.

**PARTICIPANTS:**

Thirty-three international anaesthesiology experts with recognised experience in EEG use, teaching and/or research. Experts were purposively sampled to ensure diversity in geography, clinical background, subspecialty focus and broad spectrum of perspectives.

**INTERVENTION(S):**

Not applicable.

**MAIN OUTCOME MEASURES:**

Consensus on learning outcomes, defined as at least 80% agreement (score of 9 or 10) on a 10-point Likert scale for Rounds 1 to 3, or at least 80% agreement for binary yes/no responses in Round 4.

**RESULTS:**

Consensus was achieved for 58 out of 191 learning outcomes across both basic and advanced EEG training levels. Accepted items were grouped into dimensions, including EEG Fundamentals, Raw EEG, Anaesthetic Effects, Processed EEG, Artefacts, Nociception, Patient Variability, Integration with Anaesthetic Management, Clinical Immersion and Continuous Professional Learning. Foundational dimensions had higher agreement than advanced or emerging areas, such as neurofeedback or pharmacogenomics.

**CONCLUSIONS:**

This Delphi consensus defines a comprehensive framework of EEG learning outcomes for anaesthesiology education. The framework supports the development of structured curricula and reflects a growing need for EEG competence in anaesthesia practice to improve patient monitoring and safety. Further research is needed to validate the outcomes in educational and clinical settings.

## Graphical abstract

**Figure:**
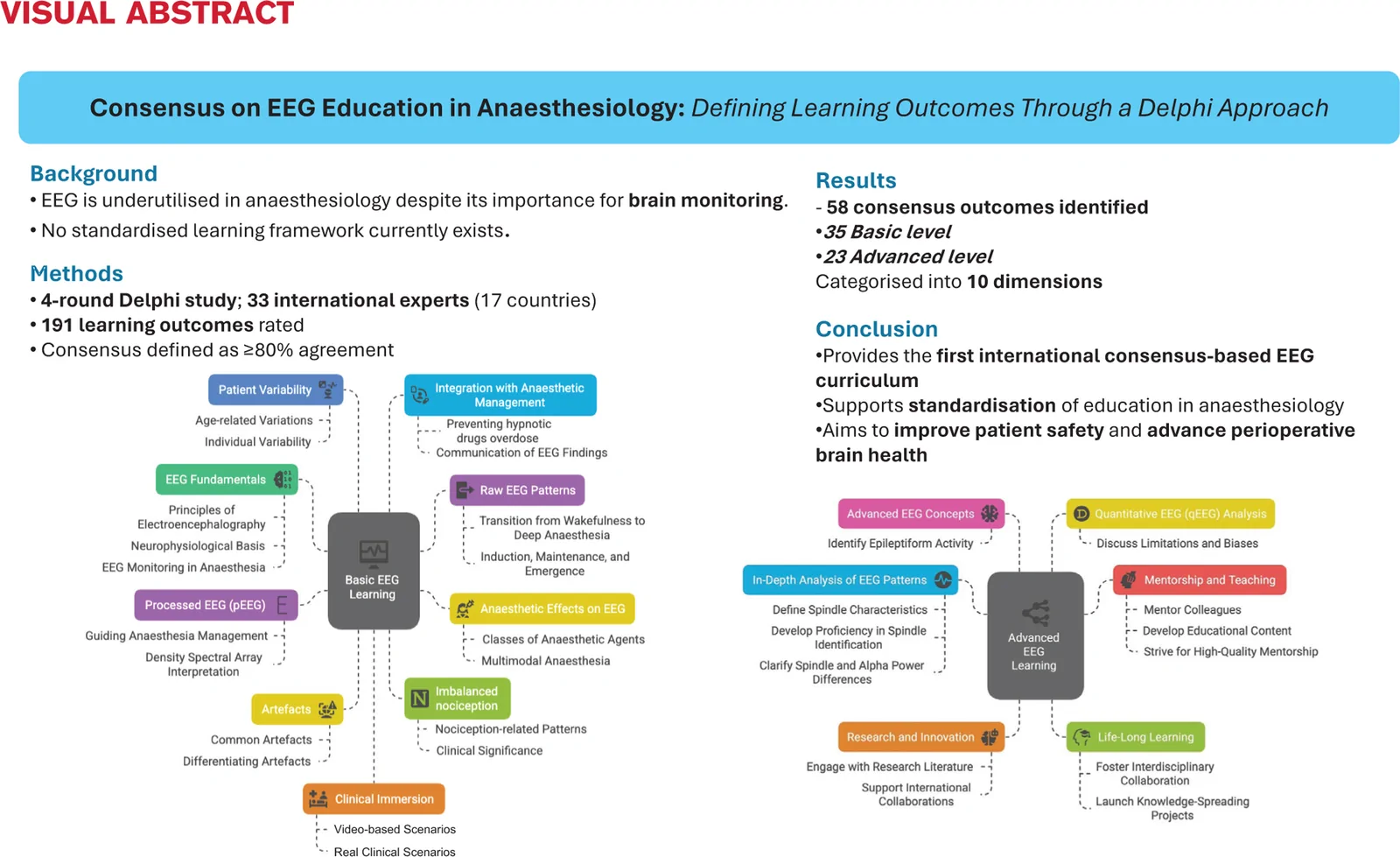

**KEY POINTS**

- Routine EEG monitoring remains underused in anaesthesia despite its potential to improve brain monitoring and patient safety.
- A multinational Delphi process involving 33 experts achieved consensus on 58 EEG learning outcomes for basic and advanced anaesthesia training.
- Core educational dimensions include EEG Fundamentals, Raw EEG Interpretation, Anaesthetic Effects, Processed EEG, Artefacts and Integration into Anaesthetic Management.
- Consensus was higher for foundational concepts than for advanced or emerging topics such as neurofeedback and pharmacogenomics.
- This framework provides a structured foundation for EEG education, supporting the development of standardised curricula in anaesthesiology.

## Introduction

The brain is the principal target organ of general anaesthesia, as the primary goal of such anaesthesia is to induce and maintain unconsciousness. Nonetheless, the brain remains the most challenging organ to monitor intra-operatively. Partly, this is because the presence and, therefore, loss of consciousness are complex concepts philosophically and scientifically difficult to quantify.^1^ In contrast, other physiological systems allow for easier monitoring through well established and quantifiable metrics such as heart rate, blood pressure, oxygen saturation and core temperature.

Despite the conceptual challenges surrounding the definition and measurement of ‘depth of anaesthesia’,^2,3^ significant progress has been made in understanding the neurophysiological underpinnings of anaesthetic-induced unconsciousness. Over the past few decades, electroencephalography (EEG) has emerged as a valuable tool for real-time assessment of the brain's electrical activity.^4–7^ Specific EEG signatures such as the frontal alpha-delta pattern and burst suppression have been identified as general indicators of anaesthetic-induced loss of consciousness.^8^ Burst suppression is now also widely recognised as an electroencephalographic marker of excessive anaesthetic depth.^9–11^

Until the mid-1990 s, the practical use of EEG in the operating room was limited by the complexity of waveform interpretation and the technical demands of recording. The introduction of commercially available processed EEG (pEEG) monitors, including the bispectral index (BIS, Covidien, Ireland) and patient state index (PSI) (Masimo, Irvine, California, USA), addressed many of these barriers.^12^ These devices convert complex EEG signals into simplified numerical indices, making brain monitoring more accessible to clinicians lacking specialised training in EEG interpretation. However, these simplified indices come with significant limitations. pEEG monitors rely on proprietary algorithms and are vulnerable to interference from muscle activity, electrical noise and artefacts. The indices can also be influenced by certain anaesthetic agents – such as ketamine and nitrous oxide – that do not suppress EEG activity in ‘typical’ ways.^13–16^ Moreover, they may fail to detect burst suppression, particularly in older adults or during low-voltage EEG states.^17^ These shortcomings have led to ongoing debate about the clinical utility of pEEG in guiding anaesthetic depth, preventing awareness and improving outcomes.^8–11,18^

Recently, the clinical focus of pEEG monitoring has shifted from preventing intra-operative awareness to avoiding excessive anaesthetic dosing, particularly in vulnerable populations. Excessive depth of anaesthesia may be linked to postoperative neurocognitive complications, including delirium and long-term cognitive decline.^19–24^ This evolving evidence has prompted changes in clinical guidelines and expert recommendations. Professional societies now advocate for EEG monitoring as a routine part of general anaesthesia.^25,26^ Rather than relying solely on pEEG indices, clinicians are encouraged to develop basic competency in interpreting raw EEG signals and to use visual tools such as the density spectral array (DSA) to enhance decision-making.^27^

Currently, education on EEG monitoring remains highly variable, with disparities in training quality across workshops, hospital programmes and conference-based courses. Notwithstanding high-quality publications^25,26^ and online resources^28,29^ offering structured learning opportunities, there is a critical need for a standardised educational framework to ensure consistency in training. To address these gaps, we convened an international multidisciplinary group of experts to collaboratively define core outcomes and educational priorities essential for integrating EEG into routine anaesthesia practice using a validated Delphi procedure.

## Materials and methods

### Ethics

The Cantonal Ethics Committee of Bern (KEK Bern) waived the need for ethics approval (BASEC-number: Req-2025-00243 on 17 February 2025. All participants gave their consent to participate. A cover letter explaining the purpose of each round accompanied all questionnaires. We followed the Guidance on Conducting and Reporting Delphi Studies (CREDES).^30^ This study followed the World Medical Association Declaration of Helsinki Ethical principles for medical research involving human subjects^31^ and complied with the Swiss Human Research Act.

### Study design and setting

We employed a modified four-round Delphi technique^32,33^ to define learning outcomes for EEG training at both basic and advanced levels. This method was chosen because it allows remote collaboration among diverse stakeholders, ensuring that input from various target group representatives is systematically gathered, synthesised and refined.^34,35^ The Delphi technique is beneficial for consolidating expert opinions into a concise and precise set of statements.^32^ The Delphi methodology operates on the principle that ‘pooled intelligence’ captures the collective insights of stakeholders.^36^

In brief, participants respond to questionnaires over multiple rounds. After each round, an external facilitator summarises the responses and provides feedback.^32,33^ This iterative process allows participants to reconsider and adjust their previous answers based on the group's evolving consensus, ultimately leading to a well defined agreement on key learning outcomes.^37^ It is a well established approach for achieving expert consensus and is widely used for curriculum development in medical education.^38–40^

### Questionnaire development and thematic structure

The Delphi process was preceded by a comprehensive search and synthesis of existing EEG learning materials, including scientific publications,^8,41–45^ commercial course syllabi, expert consensus statements^25,26^ and online modules.^27,28^ This material was reviewed and consolidated into a preliminary list of statements. To accommodate varying levels of training, we categorised the learning outcomes into two levels of complexity: Basic and Advanced. The Basic level focused on essential knowledge and interpretive skills required for the integration of EEG into routine anaesthesia practice. The Advanced level targeted learners involved in teaching EEG, research on EEG and advanced EEG-guided care (e.g. seizure detection, spindle interpretation, mentorship and innovation). Learning outcomes were grouped into distinct dimensions to support conceptual clarity and facilitate structured expert evaluation (Table 1). Each dimension captures a distinct thematic aspect of the broader construct being examined. The initial dimensions were also informed by relevant literature and expert input, and they were iteratively refined throughout the process based on participant feedback. This dimensional structure ensured that complex learning outcomes could be analysed comprehensively and consistently across rounds.

**Table 1:** Dimensions for the basic and advanced level learning outcomes

Basic EEG	Advanced
Fundamentals	Advanced EEG concepts
Raw EEG	In-depth analysis of EEG patterns
Anaesthetic effects on EEG	Pharmacogenomics of anaesthetic agents
Processed EEG	Quantitative EEG analysis
Artefacts	Neurofeedback in anaesthesia
Nociception	Neurophysiological correlates of nociception
Patient variability	Multimodal integration
Integration with anaesthetic management	Research and innovation
Clinical immersion	Mentorship and teaching
Continuous professional learning	Lifelong learning and education

### Study participants

Participants for the Delphi consensus were selected on the basis of their recognised expertise in EEG in anaesthesia. Our goal was to engage 30 stakeholders; to ensure adequate participation, 35 invitations were distributed. Thirty-three of these invitees participated in the Delphi rounds. In addition, six independent experts served as external reviewers of the final consensus but did not participate in the Delphi survey. The distinction between Delphi participants and external reviewers is stated in the Supplemental Digital File 1, http://links.lww.com/EJA/B234. Stakeholders were selected on the basis of their substantial expertise in the application, education or research of EEG and their demonstrated impact and influence on clinical practice and leadership in academic or policy discussions at both local and international levels. To ensure a broad spectrum of perspectives, we prioritised geographic and ethnic diversity, seeking representation across regions and healthcare systems. This strategy was intended to capture a wide range of theoretical frameworks, educational approaches and clinical practices, thereby enriching the validity and applicability of the consensus outcomes.

### Conflict of interest and bias mitigation

All participants were required to disclose any potential conflicts of interest before engaging in the Delphi process. This included affiliations with EEG monitoring device manufacturers, industry-sponsored research, consultancy roles or involvement in guideline development. Disclosures were reviewed independently by the study facilitators (JBE and SS) and used to assess potential biases in the interpretation and rating of statements. Participants were reminded to evaluate items from a balanced, evidence-informed perspective, irrespective of conflicts of interest.

To minimise bias, anonymised responses were used in all rounds, and no participant had access to individual-level feedback. The inclusion of geographically and professionally diverse stakeholders also balanced viewpoints and diluted potential dominance from individuals with industry ties or regional guideline influence.

### Data collection and management

We employed a modified eDelphi technique^33^ to facilitate stakeholder input. In the first round, all invited stakeholders received an email with a questionnaire. Follow-up reminders were sent every 10 days until the round was completed. Stakeholders who completed the first round were invited via email to participate in the following online rounds.

For rounds 1, 2 and 3, responses were collected using a 10-point Likert scale (ranging from 1 = strongly disagree to 10 = strongly agree). After each round, we analysed the responses by calculating the median, interquartile range [IQR], and percentage of agreement. Agreement was determined by summing responses rated 9 or 10 and estimating their proportion relative to the total responses for each statement. For round 4, participants could only answer ‘yes’ or ‘no’ to decide whether the remaining items should be included in the final list.

### Consensus definition

Although no universally accepted guidelines exist for Delphi studies, most define consensus as a specific percentage of participants agreeing on a given statement. In this study, we predefined consensus as at least 80% agreement, meaning that at least 80% of participants had to rate a statement as Strongly Agree (9 or 10) on a 10-point Likert scale. This threshold was chosen as it represents a highly rigorous level of agreement.^46^ Statements reaching consensus were included in the final list, while those with less than 50% agreement were excluded immediately.

After each round, the one facilitator (JBE), expert in medical education, reviewed all comments, refined the wording, merged similar responses and categorised them to structure the following questionnaire round. SS independently verified the results. Aggregated and anonised data were given to participants, including the median ratings and agreement levels from the previous round, and any items that had not reached consensus were re-rated. The revised questionnaire underwent further piloting, with final adjustments based on stakeholder feedback.

**Fig. 1:**
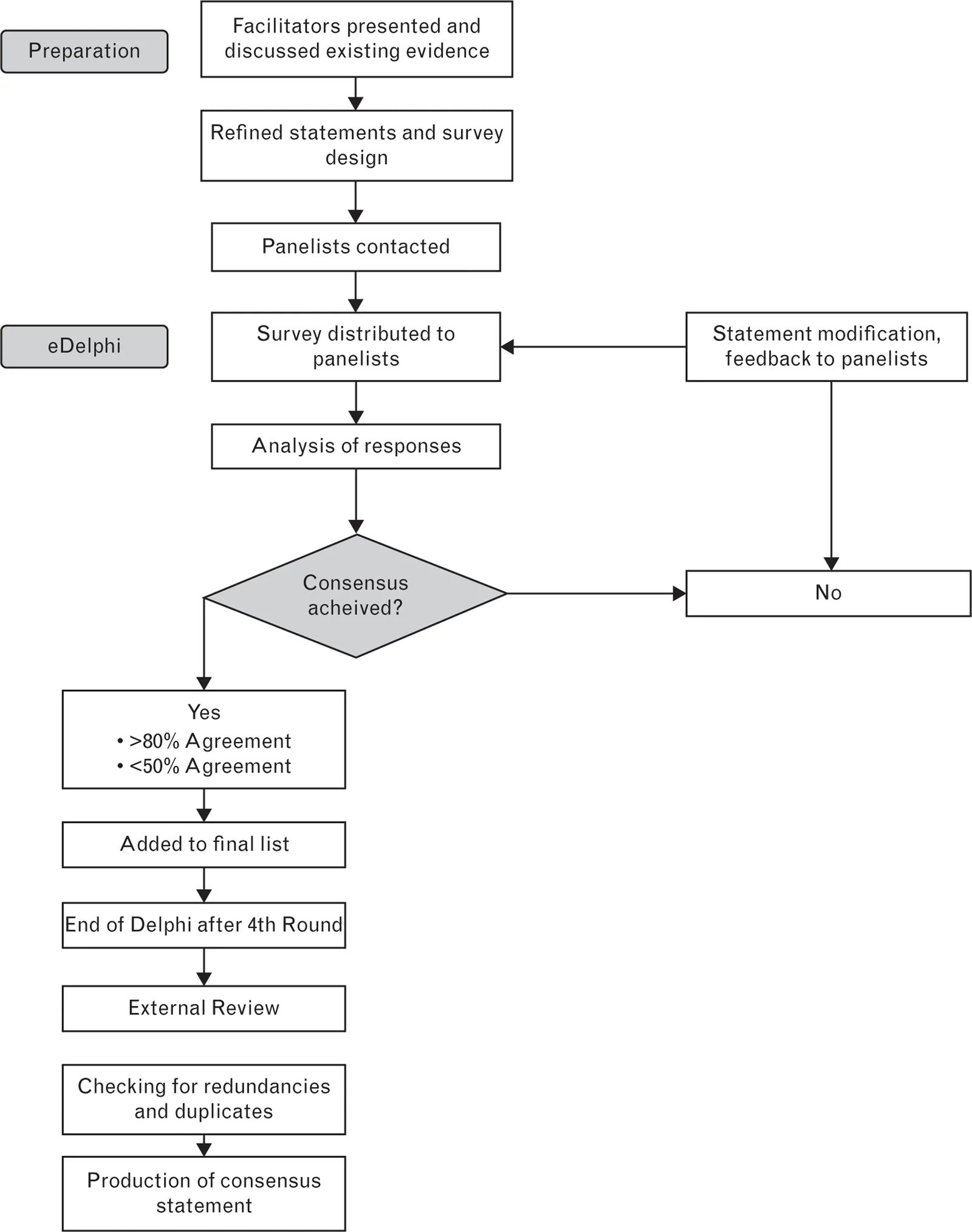
Study flowchart.

Note: In Round 4, only items with ≥80% ‘yes’ responses were accepted into the final consensus.

In round 4, participants were asked to respond with a simple yes or no to determine whether each remaining item should be included in the final list of outcomes. Only items receiving at least 80% agreement (‘yes’ responses) were accepted into the final list; all others were excluded. The prefinal list was then circulated to all stakeholders for further comments or approval. Figure 1 provides a flowchart illustrating all rounds.

Each questionnaire round was developed iteratively through continuous consultation and feedback to ensure a high-quality survey instrument. Two authors (FL and SS) pilot-tested the online version to assess the effectiveness of the questionnaire's clarity and response options. After Round 4, a final list of items was established.

In addition, an external review of the Delphi findings was conducted to ensure an impartial evaluation and validation of the consensus-building process. This external scrutiny aimed to enhance the credibility and robustness of the study outcomes by incorporating independent expert perspectives on the panel's discussions and conclusions. The involvement of external reviewers, who were not directly engaged in the Delphi rounds, provided an additional layer of objectivity and critical appraisal. This rigorous assessment strengthened the validity and reliability of the final consensus. This final version was then circulated to all members, who were asked to support or provide feedback on the document. Minor revisions were made based on the consensus at this stage. Ultimately, all recommendations were unanimously approved unless otherwise noted.

### Data handling

A descriptive analysis was performed for each round of questionnaire responses. Data from all rounds were securely stored in Google Forms, with access restricted to the facilitator (JBE) through password protection. This ensured confidentiality and maintained data integrity throughout the study.

## Results

**Fig. 2:**
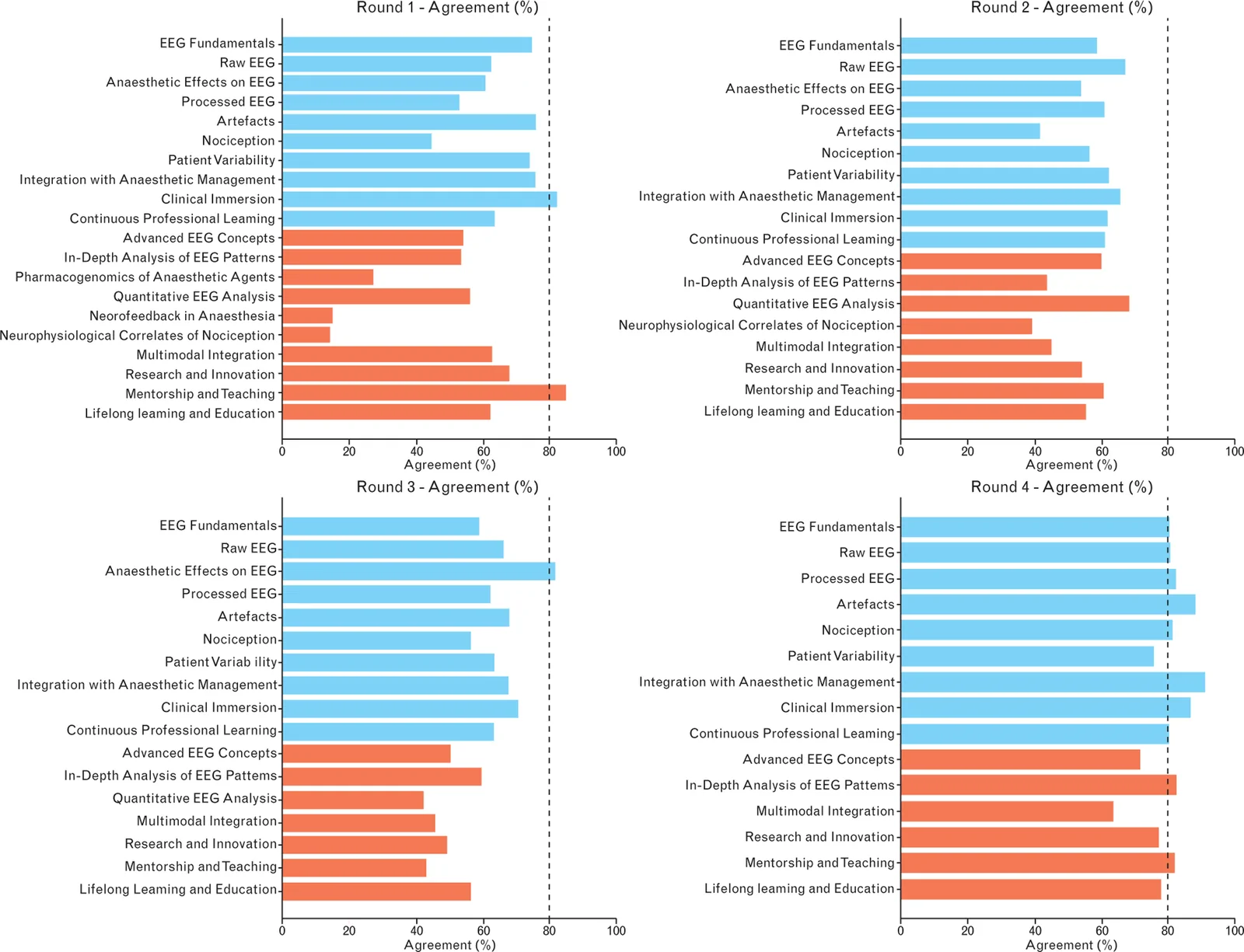
Summary of agreement and consensus levels during the different rounds.

Data were collected between 29 May 2023 and 28 November 2024. Round 1 lasted 15 days, round 2 lasted 21 days, round 3 lasted 19 days and round 4 lasted 28 days. There were no dropouts after enrolment. Figure 2 and Table S1 (Supplemental Digital File 2, http://links.lww.com/EJA/B235) depict a summary of agreement and consensus levels during the different rounds.

### First Delphi round results

The response rate was 100% (*n* = 33 participants). The median and percentage of agreement for each item are shown in Table S2 (Supplemental Digital File 3, http://links.lww.com/EJA/B236). The overall agreement for Round 1 was 58.1%. Of the 29 statements concerning Basic Level outcomes, eight (28%) were accepted as consensual and four were excluded. Seventeen items (59%) showed disagreement and were taken up in Round 2. Of the 30 statements concerning Advanced Level outcomes, three (10%) were accepted as consensual and 12 were rejected. Fifteen items (50%) showed disagreement and were taken up in Round 2. The overall agreement for each dimension is shown in Fig. 2.

### Second round results

The response rate was 100%. The median and percentage of agreement for each item are shown in Table S3 (Supplemental Digital File 4, http://links.lww.com/EJA/B237). The overall agreement for Round 2 was 56.4%. During this round, 122 learning outcomes were added for analysis.

For the basic learning outcomes, we analysed 96 statements, of which 25 (26%) were accepted as consensual and 31 were excluded. Forty statements (42%) showed disagreement and were taken up in Round 3. The overall agreement for each dimension is shown in Fig. 2.

As for the advanced learning outcomes, 58 statements were analysed in Round 2. Of those, two (3%) were accepted as consensual. Twenty-eight statements were rejected, and 28 (48%) showed disagreement and were taken up in Round 3.

### Third round results

The response rate was 100%. Table S4 (Supplemental Digital File 5, http://links.lww.com/EJA/B238) shows each item's median and percentage of agreement.

We analysed 51 statements regarding the basic learning outcomes, of which eight (16%) were consensual. The overall agreement for Round 3 was 59.1%. The agreement for each dimension is shown in Fig. 2. We excluded six statements. Thirty-seven statements (73%) showed disagreement and were taken up in Round 4.

None of the 27 advanced-level statements analysed in Round 3 were accepted as consensual. Twelve statements were rejected, and 15 (56%) showed disagreement and were taken up in Round 4.

### Fourth round results

Stakeholders assessed the remaining 52 sentences for inclusion in the final document. The response rate was 100%. Sentences with an 80% or more agreement were selected for the final listing. This round reached an acceptance consensus for 31 items (57%). Overall agreement was 79.4%. Table S5 (Supplemental Digital File 6, http://links.lww.com/EJA/B239) shows all items included and excluded in this round.

### External review

**Fig. 3:**
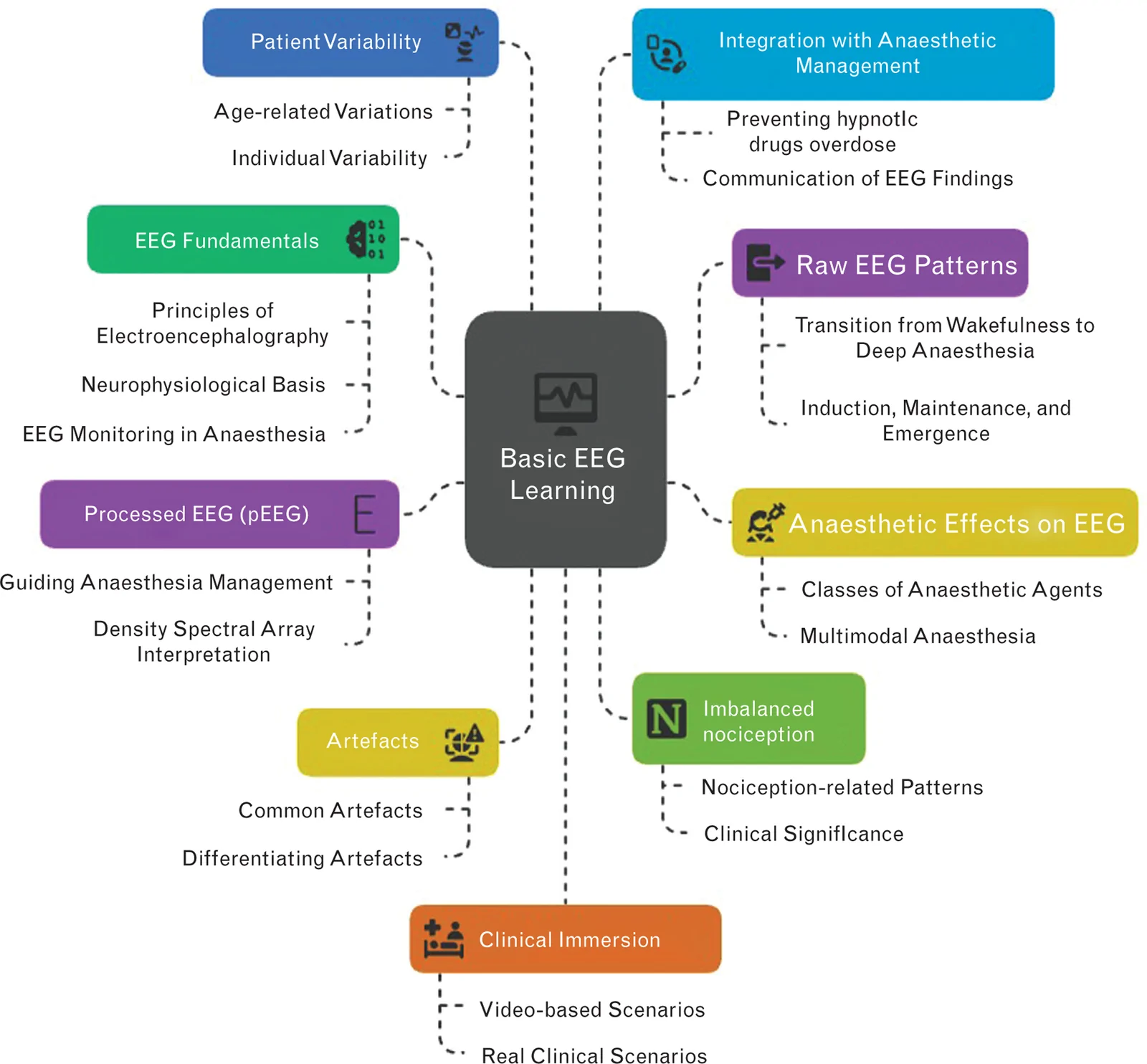
Educational framework for basic EEG learning in anaesthesia.

**Fig. 4:**
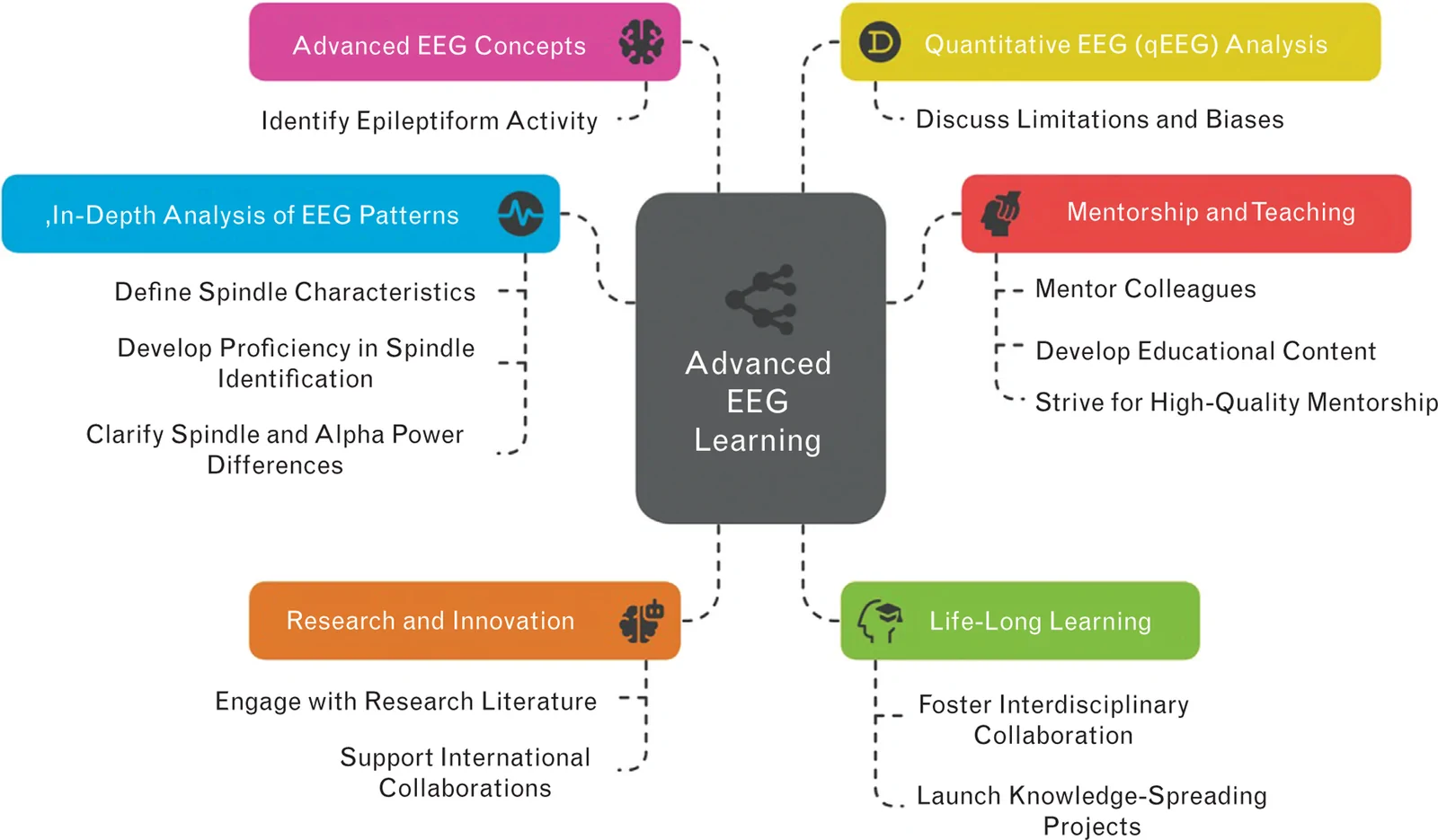
Educational framework for advanced EEG learning in anaesthesia.

We followed NASA's recommendation^32,38^ to have our results externally reviewed upon completion of data handling. Five anaesthesiology experts (AA, BP, SS, MG, CR) and one trainee (IC) were selected as external reviewers to comment on the validity and usefulness of our methodology and results. The criteria for selection included a track record of scholarly contributions, experience in clinical practice and expertise in research methodology relevant to the study. These external reviewers evaluated the methodology, data handling and validity of the study findings according to a checklist (Supplemental Digital File 7, http://links.lww.com/EJA/B240) and assessed their practical usefulness. After merging redundant outcomes and removing duplicates (Table S6, Supplemental Digital File 8, http://links.lww.com/EJA/B241), 58 statements out of 191 assessed learning outcomes remained. This framework defines the core outcomes and educational priorities for the Learning and Teaching of EEG for Anaesthesiologists (Tables 2 and 3, Figs. 3 and 4).

**Table 2:** Basic level learning outcomes achieving consensus in round 4, organised by dimension

EEG Fundamentals	
FD1	To describe the basic principles of electroencephalography (EEG)
FD2	To describe the neurophysiological basis of EEG signals
FD3	To explain why EEG monitoring is applied in the context of anaesthesia
FD4	To demonstrate proficiency in the correct application of EEG electrodes
FD5	To identify and troubleshoot common technical issues related to EEG monitoring
FD8	To name the clinical indications for EEG monitoring and their importance
FD12	To identify and recognise basic EEG patterns relevant to anaesthesiology
FD13	To describe the relationship between EEG signal signatures and clinical effects
FD14	To outline key aspects of EEG hardware, including electrodes, impedance and filters
FD19	To explain the importance of recognising basic EEG patterns for anaesthesiologists
Raw EEG	
RW4	To identify and explain the fundamental raw EEG patterns.
RW5	To demonstrate the transition of EEG patterns from wakefulness to deep anaesthesia, highlighting changes in amplitude and peak frequency.
RW7	To recognise, discuss and interpret raw EEG patterns associated with different phases or stages of anaesthesia (induction, maintenance, and emergence), including the baseline raw EEG before anaesthesia.
RW11	To recognise and differentiate spike and burst suppression patterns in EEG recordings.
RW12	To identify changes in raw EEG patterns associated with various types of anaesthetic agents.
RW13	To analyse and evaluate the feasibility of assessing different stages or phases of anaesthesia using raw EEG, emphasising the effectiveness of raw EEG in awake, alpha-delta anaesthesia and burst suppression.
Anaesthetic effects on the EEG	
EF1	To identify how different classes of anaesthetic agents contribute to distinct EEG signatures.
EF7	To differentiate between single-agent EEG signatures and how multimodal anaesthesia, including drug combinations, can affect EEG patterns.
(Semi-)Processed EEG (pEEG)	
PR2	To interpret and use the pEEG to guide anaesthesia management
PR3	To describe the basic principles of spectral analysis and the interpretation of the Density Spectral Array (DSA)
PR4	To describe the conceptual aspects of pEEG, focusing on reading and interpreting spectral analysis rather than detailed index creation (e.g. BIS).
PR5	To recognise the strengths and limitations of using pEEG compared to raw EEG
PR6	To discuss the relevance of parameters such as Spectral Edge Frequency (SEF), EMG (Electromyogram), Burst Suppression Ratio (BSR) and Signal Quality Index (SQI)
PR14	To critique the use of EEG indices and discuss their potential unreliability and risk of misinterpretation.
Artefacts	
AR1	To identify common artefacts in EEG recordings (EMG, electrode movement, electrical noise, eye movements).
AR2	To apply techniques to differentiate artefacts from genuine EEG signals
AR3	To demonstrate proficiency in recognising and mitigating artefacts during EEG monitoring.
AR5	To distinguish between technical and physiological artefacts
Nociception	
NC11	To recognise and describe nociception-related EEG patterns and understand their significance in clinical practice
NC12	To interpret EEG changes, such as alpha drop-out, in conjunction with other clinical signs and events, emphasizing the importance of a comprehensive clinical assessment.
Patient variability	
PV1	To appreciate variations in EEG features in specific patient populations (older adults, paediatric).
PV2	To describe how age-related and developmental differences impact EEG monitoring
PV3	To adjust anaesthesia management based on patient-specific EEG variations
PV6	To recognise the importance of individual variability beyond age-related differences in anaesthesia management and EEG interpretation.
PV8	To develop a fundamental understanding of concepts such as ‘frequency-band-power’ and frontal alpha-waves in EEG monitoring.
PV10	To explore the outcomes associated with burst suppression patterns
PV11	To learn how to identify high-risk patients for postoperative neurocognitive dysfunction (PND) through pre-operative or intra-operative EEG assessment
Integration with anaesthetic management	
AM1	To discuss how EEG monitoring integrates with overall anaesthetic management
AM2	To discuss the role of EEG data in preventing over-sedation or inadequate anaesthesia
AM3	To demonstrate effective communication of EEG findings to the anaesthesia team to inform decision-making.
Clinical immersion	
CI1	To engage in clinical immersion experiences, including video-based scenarios, to observe and interpret real-world EEG patterns during anaesthesia.
CI2	To emphasise the importance of using video and pictures from real clinical scenarios to enhance learning
CI5	To use quizzes for recognition of artefacts and medications to reinforce learning
Continuous professional learning	
PL1	To cultivate a commitment to continuous learning and stay updated on advancements in EEG monitoring technology and practices
PL3	To foster an interest in EEG monitoring
PL4	To establish continuous update sessions or mechanisms for ongoing education and skill development

**Table 3:** Advanced level learning outcomes achieving consensus in round 4, organised by dimension

Advanced EEG concepts	
AC7	To identify epileptiform activity in the EEG
In-depth analysis of EEG patterns	
AN1	To define the characteristics of spindles in EEG patterns, including frequency range, amplitude and typical locations on EEG channels.
AN4	To develop proficiency in visually identifying spindle patterns on EEG recordings.
AN12	To clarify terminological differences between spindle activity and alpha power
Quantitative EEG (qEEG) analysis	
QT1	To discuss the limitations and potential biases associated with quantitative EEG metrics
Research and innovation	
RI1	To engage with current research literature on advanced EEG applications in anaesthesia
RI8	To support international collaborations in EEG research, encouraging a global exchange of knowledge and data
Mentorship and teaching	
MT1	To mentor colleagues and contribute to their understanding of advanced EEG concepts
MT2	To develop and deliver educational content on advanced EEG monitoring for anaesthesia
MT4	To strive for high-quality mentorship and teaching in the use of EEG
Life-long learning	
LL2	To foster interdisciplinary collaboration with neuroscientists, engineers and other healthcare professionals to advance the field of EEG monitoring in anaesthesia
LL6	To launch projects aimed at spreading knowledge about EEG in anaesthesia, simplifying the learning of basics, and developing comprehensive knowledge, teaching methodologies and management protocols

## Discussion

This study represents the first consensus process explicitly defining structured EEG learning outcomes for anaesthesia education at both basic and advanced levels. The exceptionally high engagement rate underscores the strong interest and commitment of experts in the field to advancing EEG integration in anaesthesia practice while also reflecting widespread recognition of the existing gap in clinical implementation and education. Through rigorous methodology, consensus was achieved on 58 learning outcomes, forming a comprehensive framework for EEG education. In terms of clinical relevance, this framework addresses a longstanding educational deficiency in anaesthesiology training, aligning competencies with evolving practice standards and patient safety priorities. Traditional teaching has focused primarily on the prevention of intra-operative awareness.^47^ However, current knowledge has identified a need for avoiding excessive anaesthetic depth through monitoring, given its growing association with postoperative neurocognitive complications, including delirium and long-term cognitive decline.^25,26^ The defined outcomes reflect this paradigm shift, offering a structured foundation for training that supports well tolerated, evidence-based and individualised anaesthesia care. Notably, the framework aligns with evolving clinical guidelines and advocates for broader EEG use, particularly in vulnerable patient populations such as older adults or those at a high risk for PND.^25,26^

The variation in agreement across thematic dimensions probably reflects differing levels of clinical relevance and evidence supporting each topic. The strong consensus on ‘Anaesthetic Effects on EEG’ suggests this area is well established, widely taught and considered essential for clinical practice. In contrast, the lower agreement on ‘Nociception’ and ‘Neurofeedback’ suggests that the application of EEG for these topics/dimensions is considered more experimental and less standardised in anaesthetic practice.

The high number of rejected items – particularly within advanced and theoretical dimensions – suggests that while these concepts may be intellectually valuable or hold future potential, they are not yet perceived as essential or feasible for inclusion in core clinical educational frameworks. This reflects a pragmatic approach, prioritising actionable, teachable and directly relevant outcomes to current clinical practice over more speculative or research-oriented content. These rejections provide valuable insights for curriculum developers. They emphasise the need to focus on clinically impactful and evidence-based content. These findings support a tiered approach to EEG education, focusing on universally accepted fundamentals for all anaesthesia trainees while gradually incorporating more advanced or debated topics as the evidence base and andragogical strategies continue to evolve.

This study has several strengths. It comprehensively covered EEG-related topics, from basic principles to advanced clinical applications. The strong consensus in dimensions such as EEG Fundamentals and Raw EEG underscores the recognition of foundational knowledge as crucial for well tolerated and effective anaesthetic management. In addition, the high agreement in pEEG and Integration with Anaesthetic Management highlights the growing importance of advanced monitoring techniques and their integration into clinical decision-making. These findings support the development of a structured EEG curriculum tailored to anaesthesia, bridging the gap between theoretical knowledge and practical application.

However, despite the high level of agreement in many areas, specific dimensions showed substantial disagreement, necessitating multiple rounds for consensus. Specifically, advanced topics such as ‘Neurofeedback in Anaesthesia’ and ‘Pharmacogenomics of Anaesthetic Agents” exhibited lower agreement levels, indicating the evolving nature of these fields and the need for further research. The disagreement in these areas may reflect variability in clinical experience, diverse perspectives on the applicability of cutting-edge EEG technologies or a lack of standardised guidelines.

The study also has limitations. Firstly, its reliance on expert consensus, which is subjective and can be influenced by group dynamics. It may not fully capture the perspectives and experiences of the broader anaesthesia community on educational needs. Secondly, the study's findings are based on consensus rather than empirical validation. This limits the generalisability and practical applicability of the results. Thirdly, a significant limitation is the ongoing rapid evolution of EEG technology. The consensus in this study may become outdated as new research and clinical practices emerge. This underscores the need for continuous updates to the educational framework to maintain its relevance and accuracy.

Furthermore, although we aimed to include geographically diverse participants, the participants may not fully represent the global anaesthesiology community. Cultural differences, resource availability and regional variations in clinical practice could influence perspectives on EEG monitoring. The study also faced challenges related to the complexity of advanced topics. For example, dimensions associated with advanced EEG concepts and neurophysiological correlates of nociception showed substantial disagreement, reflecting the evolving nature of these fields and the need for further research. In addition, the study did not assess the feasibility of implementing the proposed educational framework in clinical settings, as it did not consider resource limitations, time constraints, and the availability of trained educators. Moreover, the historical reliance on pEEG indices and existing scepticism about their utility may have biased participants’ opinions, influencing the consensus on more advanced or nuanced applications of EEG monitoring. Finally, although the study successfully defined learning outcomes, it did not validate whether achieving them would improve clinical decision-making or patient outcomes.

This Delphi study provides a comprehensive and structured framework for EEG education in anaesthesia, balancing foundational knowledge with advanced applications. The findings highlight the need for a tiered educational approach, reflecting varying levels of complexity and clinical relevance. By achieving consensus on 58 EEG learning outcomes, this study lays the groundwork for developing standardised curricula and guidelines for EEG teaching and, ultimately, for enhancing patient safety and clinical decision-making in anaesthesia practice.
